# Risk factors for infection following prostate biopsy - a case control study

**DOI:** 10.1186/s12879-015-1328-7

**Published:** 2015-12-23

**Authors:** Elliot Anderson, Olivia Leahy, Allen C. Cheng, Jeremy Grummet

**Affiliations:** Monash University, Melbourne, Victoria Australia; Department of Urology, Alfred Health, Melbourne, Victoria Australia; Department of Epidemiology and Preventative Medicine, Monash University, Melbourne, Victoria Australia; Department of Infectious Diseases, Alfred Health, Melbourne, Victoria Australia; Department of Surgery, Monash University, Melbourne, Victoria Australia

**Keywords:** Prostate, Needle biopsy, Transrectal biopsy, Infection, Sepsis, Case control

## Abstract

**Background:**

Infection is a complication of TRUS prostate biopsy, despite the use of antimicrobial prophylaxis. Worryingly the rate of infectious complications following TRUS biopsy has been shown to be increasing. We aimed to determine the rate, severity, risk factors, standard patterns of care and microbiology resistance profiles associated with TRUS biopsy sepsis.

**Methods:**

A retrospective case–control study was conducted. Using electronic coding all patients who presented to Cabrini Hospital with sepsis following a TRUS biopsy from 2009 to 2013 were identified. Validated cases were matched to controls in a ratio of 1:3. Eligible controls were required to have undergone a TRUS biopsy at the same surgical institution as the case and in the closest period of time. Demographic, procedural and patient related data-points were recorded for all patients using hospital and urologist records. Univariate logistic regression models were constructed and used to determine risk factors associated with infection.

**Results:**

71 cases developed sepsis following TRUS biopsy and were matched to 213 controls. The average rate of sepsis over the 5-year study period was 1.5 %. A SOFA score ≥ 2 was identified in 28 % of cases. We found a high prevalence of antimicrobial resistant E. coli, with 61 % of blood culture isolates classified as Multidrug resistant organisms. Eight different prophylactic antimicrobial regimens were identified with 33 % of cases receiving ineffective antimicrobial prophylaxis. Statistically significant risk factors included previous antimicrobial use and prior international travel within the six months prior to biopsy.

**Conclusions:**

TRUS biopsy is an elective procedure and as such needs to be associated with minimal morbidity. The patterns of care surrounding periprocedural variables for TRUS biopsies were non-uniform and diverse. A wide variety of different prophylaxis regimens and bowel preparation routines were recorded. Patients with risk factors for sepsis may represent a better target population for intervention with alternative preventative strategies. Alternative preventative options include augmented prophylaxis, tailored prophylaxis or the TP biopsy approach either as a first line biopsy modality or based on epidemiological risk factors.

## Background

The diagnosis of prostate cancer is confirmed by prostate biopsy. Transrectal ultrasound-guided (TRUS) biopsy is the most common method of obtaining prostate tissue for analysis [1]. TRUS biopsies are typically considered to be a well-tolerated outpatient procedure however complications are well recognized [2]. Current practice guidelines unanimously recommend the use of antimicrobial prophylaxis prior to a TRUS biopsy, which has been shown to significantly decrease the risk of post-biopsy infectious complications [3–5]. However infectious complications may still develop with the reported rate of sepsis ranging from 0.6-5.7 % in contemporary studies [6, 7] with evidence of an increasing trend [8, 9].

The proposed pathophysiology of post-biopsy infection most likely results from the transrectal passage of the biopsy needle. This approach allows for the inoculation of bacteria from the rectal mucosa directly into the prostate, blood vessels or urinary tract [10, 11]. The best evidence suggests that the presence of endogenous antimicrobial-resistant bacteria is a significant risk factor for infectious complications [12–15]. In particular, Steensels et al. found that the presence of endogenous fluoroquinolone-resistant bacteria in men undergoing a TRUS biopsy was a risk factor for developing post-biopsy infectious complications [16]. The risk of acquiring endogenous antimicrobial-resistant bacteria may be increased by several factors such as antimicrobial use [17–20] or international travel prior to TRUS biopsy [20, 21].

Other risk factors for developing sepsis following TRUS biopsy can be categorized as patient-related or procedural. No definitive patient or procedure-specific risk factors have been identified, despite analysis of patient co-morbidities [9, 22, 23], the use of pre-biopsy enema [6, 24] or the number of cores taken during the procedure [25].

In the present study we aimed to determine the rate, severity, risk factors and microbiology resistance patterns associated with TRUS biopsy sepsis. As well as the standard patterns of care surrounding TRUS prostate biopsy.

## Methods

The population was defined as patients undergoing a TRUS prostate biopsy by a urologist associated with Cabrini Hospital, a private 700-bed metropolitan hospital in Melbourne. This included patients who had their TRUS biopsy at smaller neighbouring institutions, but in the event of complications were instructed to present to Cabrini Hospital’s Emergency Department. Neighbouring institutions included Linacre Private Hospital, Masada Private Hospital and the Avenue Hospital. All biopsies were completed using a standard ultrasound and biopsy probe with patients receiving prophylactic antimicrobials and intravenous sedation prior to their procedure. A case was defined as a patient who presented to hospital with sepsis due to suspected or confirmed infection related to the genitourinary tract or where no other focus of infection was clinically evident within 14 days of undergoing a TRUS biopsy from 2009 until 2013. Sepsis was defined according to the ACCP/SCCM (1991) criteria as a systemic inflammatory response syndrome in the presence of an infective process [26]. Patients were excluded if they underwent TRUS biopsy at a non-participating institution. Cases were identified via an electronic search of the Cabrini Health data warehouse using *Medicare Benefit Schedule* (MBS) codes for TRUS biopsy and *International Classification of Disease and Related Health Problems, Tenth Revision, Australian Modification (ICD-10-AM)* diagnosis codes for infective complications. These codes were used to screen for sepsis, which was then confirmed by a review of the patients’ medical records. Confirmed cases that underwent their TRUS biopsy at a participating institution were matched to three control patients. Eligible controls were the next patients that underwent TRUS biopsy at the same institution as the case but whom did not develop infectious complications.

Patient data, including demographic, procedural and patient-related factors were collected on a standardized form using hospital and urologist records. Where the procedure was performed at another institution, procedural data was extracted from urologists’ medical records. A history of travel was defined as international travel within six months prior to biopsy. Antimicrobial use was defined as oral or intravenous antimicrobial therapy within the six months preceding biopsy. If no history was documented, it was assumed the patient had not travelled or taken antimicrobials. We used a modified Sequential Organ Failure Assessment Score (SOFA) to determine the severity of illness. The SOFA score is a standardised assessment tool used to determine the degree of sepsis related organ dysfunction. It is calculated from the total sum of six organ system categories, each graded from 0 to 4 based on predefined measurement values. As many patients did not have an arterial blood gas, we used *arterial oxygen saturation measured by a pulse oximeter* (SpO2) as a surrogate marker for *partial pressure of arterial oxygen* (PaO_2_) [27].

To define risk factors associated with infection, we constructed univariate conditional logistic regression models, which accounted for the matched nature of the control selection. A multiple variable analysis was not performed due to the relatively low proportion of patients with any one exposure (and therefore limited statistical power) and the likelihood of co-linearity between exposures. We defined statistical significance as *p* < 0.05. As this was an exploratory study, we did not adjust for multiple hypothesis testing. Ethics approval was obtained from the Cabrini Human Research Ethics Committee.

## Results

Over the five-year study period, 71 patients required admission to Cabrini Hospital for sepsis following TRUS biopsy and were matched to 219 controls. The mean proportion of patients with sepsis across the five-year study period was 1.5 %, with the lowest rate of 0.6 % reported in 2011 and the highest rate of 2.9 % reported in 2013. No statistically significant difference regarding the rate of sepsis over the study period was noted (*p* = 0.091).

The mean duration of admission was 4.5 days (median 4 days, IQR 3,5 days, range 1–14 days). Of the 60 (76.9 %) cases where data was available to assess a SOFA score, 43 (71.7 %) patients had a SOFA score ≤1, 10 (16.7 %) patients had a score of two, 4 (6.7 %) had a SOFA score of three and 3 (5 %) patients had a SOFA score ≥4.

Urine culture results were available for 66 patients. Culture results were positive for 28 (42.4 %) patients. Isolated organisms included 27 *Escherichia coli* (96.4 %) and one *Acinetobacter* spp. (3.6 %). Of all urine isolates, 22 (78.6 %) were resistant to more than one antimicrobial agent. Blood culture results were available for 65 patients. Culture results were positive for 36 (55.4 %) patients. The organisms isolated were *E. coli* 34 (94.4 %), *Streptococcus pneumoniae* 1(2.9 %) and *Bacteroides uniformis* 1(2.9 %). Of all blood isolates 28 (77.7 %) were resistant to more than one antimicrobial agent (Table 1).

**Table 1:** Antimicrobial resistance in breakthrough blood/urine isolates

Antimicrobial class	Antimicrobial name	Urine culture (%)	Blood culture (%)
Aminoglycosides	Gentamicin	15 (53.6)	20 (58.8)
	Tobramycin	3 (10.7)	14 (41.2)
Cephalosporins	Cefepime	0	4 (11.7)
	Ceftriaxone	4 (14.3)	6 (17.6)
	Cephalexin or Cephazolin	8 (28.6)	12 (35.2)
Carbapenems	Imipenem	0	0
Fluoroquinolone	Ciprofloxacin or Norfloxacin	22 (78.6)	23 (67.6)
Nitrofurans	Nitrofurantoin	2 (7.1)	0
Penicillins	Ampicillin or Amoxycillin	18 (64.3)	24 (70.6)
	Amoxycillin/clavulanic acid	8 (28.6)	20 (58.8)
	Piperacillin	3 (10.7)	10 (29.4)
	Ticarcillin/clavulanate	6 (21.4)	8 (23.5)
Sulfonamides	Trimethoprim	17 (60.7)	2 (5.9)
	Trimethoprim/sulfamethoxazole	6 (21.4)	11 (32.4)
	Total isolate organisms^a^	28	34

^a^Total urine culture organisms: E. coli (*n* = 27), Acinetobacter spp. (*n* = 1), Total blood culture organisms: E. coli (*n* = 34)

Patient risk factors that were found to be significantly associated with the development of sepsis following TRUS biopsy included a history of international travel or recent antimicrobial use in the six months prior to a patient’s biopsy (Table 2). Other variables such as patient comorbidities, recent hospitalization, age or being a hospital employee were not significantly associated with sepsis.

**Table 2:** Risk factors for sepsis

	Cases	Controls	OR (95 % CI)	*P* value
Number	71	213		
Patient variables				
Age > 65 years	35 (49.3 %)	92 (43.2 %)	1.34 (0.74, 2.40)	0.33
Hospital employee	5 (7.0 %)	13 (6.1 %)	1.16 (0.41, 3.33)	0.78
Autoimmune condition	0 (0 %)	10 (4.5 %)	Undefined	ND
Immunosuppression	1 (1.4 %)	3 (1.4 %)	1.00 (0.08, 11.93)	1.00
COPD	2 (2.8 %)	4 (1.9 %	1.50 (0.27, 8.19)	0.64
Heart valve replacement	3 (4.2 %)	3 (1.4 %)	3.0 (0.61, 14.86)	0.18
Benign prostate enlargement	14 (19.7 %)	41 (19.2)	1.24 (0.62, 2.43)	0.54
Diabetes	10 (14.1 %)	23 (10.8 %)	1.35 (0.61, 2.96)	0.46
Recent travel	13 (18.3 %)	7 (3.2 %)	5.40 (1.61, 18.09)	0.01
Recent antimicrobial use	7 (9.8 %)	3 (1.4 %)	9.59 (1.97, 46.62)	0.01
Recent hospitalisation	1 (1.4 %)	0 (0.0 %)	Undefined	ND
Procedural variables				
Use of FQ prophylaxis	70 (98.6 %)	213 (100.0 %)	Undefined	ND
Duration of FQ prophylaxis <7 days	10 (14.1 %)	44 (20.7 %)	1.72 (0.71, 4.20)	0.23
Use of aminoglycoside prophylaxis	19 (26.8 %)	43 (20.2 %)	1.85 (0.81, 4.24)	0.14
Use of a penicillin as prophylaxis	19 (26.8 %)	76 (35.7 %)	0.42 (0.18, 0.98)	0.05
Use of carbapenem as prophylaxis	0	21 (9.8 %)	Undefined	ND
Previous biopsy	12 (16.9 %)	36 (16.9 %)	1.29 (0.62, 2.70)	0.50
Previous biopsy within 3 years	11 (15.5 %)	26 (12.2 %)	1.31 (0.61, 2.83)	0.48
Urinary catheter	0 (0 %)	0 (0 %)	Undefined	ND
Preoperative urine culture	1 (1.4 %)	0 (0 %)	Undefined	ND
Use of enema	31 (43.6 %)	110 (51.6 %)	0.71 (0.30, 1.69)	0.44
Prostate volume >30 mL	53 (74.6 %)	174 (81.7 %)	1.38 (0.64, 2.93)	0.41
PSA > 4 mmol	55 (77.4 %)	168 (78.8 %)	Undefined	ND
Number of biopsy cores >12	46 (64.8 %)	156 (73.2 %)	0.37 (0.14, 0.95)	0.04
Prostate cancer	42 (59.2 %)	123 (57.7 %)	1.15 (0.63, 2.07)	0.65

*ND* not done

The number of biopsy cores most commonly taken was 14 [Range: 7,22]. More than 12 cores were taken in 63 (88.7 %) case cohort patients and in 202 (94.8 %) of control cohort patients. Patients who did not develop sepsis were significantly more likely to have had more than 12 biopsy cores taken (OR 0.37, 95 % CI 0.14, 0.95, *p* = 0.04). Other procedural variables such as the use of a pre-biopsy enema, prior TRUS biopsy, prostate volume or biopsy result were not shown to be significantly associated with infection.

All patients in this study received antimicrobial prophylaxis. Over the course of the study eight different antimicrobial regimens were identified (Table 3). Seven of the identified regimens included the use of a fluoroquinolone antimicrobial and this accounted for 282 (99.3 %) men. The most common prophylaxis regimen was the use of a fluoroquinolone agent alone, followed by a fluoroquinolone antimicrobial in combination with a penicillin antimicrobial. All prophylactic regimens were distributed equally across the study period. This study found that the addition of a penicillin antimicrobial (amoxicillin) to standard fluoroquinolone prophylaxis resulted in a reduced risk of developing sepsis (OR 0.42, 95 % CI 0.18, 0.98, *p* = 0.05). However, when this risk was calculated without carbapenem co-administration, 26.8 % of cases and 31.5 % of controls developed sepsis suggesting pencillins were not effective. There was also shown to be no significant difference between the use of three days or seven days of fluoroquinolone antimicrobials (OR 1.72, 95 % CI 0.71, 4.20, *p* = 0.23) No patient who received a carbapenem antimicrobial prophylactically developed sepsis following a TRUS biopsy.

**Table 3:** Prophylactic antimicrobial regimens used

Antimicrobial class^a^	# Cases (%)	# Controls (%)
F	28 (39.4)	88 (41.3)
F + P	18 (25.3)	63 (29.5)
F + P + A	1 (1.4)	4 (1.8)
F + A	23 (32.3)	37 (17.3)
F + P + C	0 (0)	8 (3.7)
F + P + A + C	0 (0)	1 (0.4)
F + C	0 (0)	11 (5.1)
C + A + CE	0 (0)	1 (0.4)
Unknown	1 (1.4)	0 (0)
Total	71	213

^a^ *F* fluoroquinolone, *P* penicillin, *A* aminoglycoside, *C* carbapenem, *CE* cephalosporin

## Discussion

In this study, we found that the rate of post-biopsy sepsis was 1.5 % across the five-year study period. This is consistent with the intervention arms of the original clinical trials supporting prophylaxis [4], and may reflect relatively low rates of fluoroquinolone resistance in Australia [28]. This is in contrast to studies in North America that have demonstrated a rising rate of post-biopsy sepsis most likely attributable to increasing bacterial resistance [8, 9]. In addition we found that a third of septic patients had evidence of significant organ dysfunction, consistent with severe sepsis, although we did not observe any mortality. This is in line with previous studies suggesting the mortality from prostate biopsy is low [8, 9].

As in similar studies, E. coli was the predominant causative organism in our series, accounting for 95 % of positive cultures [13, 25, 29, 30]. A concerning trend has been the development and spread of genes providing resistance to beta-lactams, quinolones and aminoglycoside antimicrobials [31]. Fluoroquinolone resistance has been linked to failure of prophylaxis in previous studies [16, 17]. Notably in our series, 68 % and 59 % of breakthrough blood culture isolates were resistant to fluoroquinolones and gentamicin respectively. However, we found that only a third of cases received an ineffective prophylaxis regimen, based on resistance patterns of isolated organisms. This suggests that factors other than antimicrobial resistance may be responsible for the failure of prophylaxis, and that switching from fluoroquinolones to aminoglycosides may not necessarily be more effective. The proportion with fluoroquinolone resistance is similar to that found in a New Zealand study [32] but lower than in North American studies [14].

A wide diversity of antimicrobial prophylaxis regimens has been reported in the literature [33–37]. This study identified eight different prophylaxis protocols over the study period reflecting considerable clinical uncertainty. The most effective prophylactic regimens include carbapenem antimicrobials with this study supporting Losco et al’s finding of a 0 % sepsis rate amongst men undergoing a TRUS biopsy [38]. Although carbapenem antimicrobials appear to be highly effective, they can only be administered parenterally, and there is concern that widespread use may result in the development and spread of carbapenem-resistant Enterobacteriaceae (CRE) [38, 39]. Other strategies to minimize risk include the use of alternative prophylactic agents (e.g. nitrofurantoin and/or fosfomycin), [40, 41] the use of targeted prophylaxis based on microbiological screening for resistance, or alternative surgical approaches, such as transperineal biopsy.

Consistent with other reports, we found that international travel in the six months prior to the procedure was associated with post-biopsy sepsis. Although this association may be over-estimated due to recall bias, it is known that the prevalence of fluoroquinolone-resistance organisms varies around the world [28], that travel is associated with carriage of antimicrobial resistant bowel flora [21] and that travel to countries with high fluoroquinolone-resistance has been shown to be a risk factor for developing sepsis following TRUS biopsy [20, 42]. Interestingly, we found that all patients who developed sepsis and travelled internationally had journeyed to the South-East Asia or Western Pacific regions. This may suggest that the risk profile of the destination may be useful in assessing risk, and help identify a group where alternative strategies or antimicrobial agents may be warranted.

This study had a number of limitations. In this observational study, we cannot exclude the possibility of unmeasured confounders. This was particularly the case as a large number of different prophylaxis regimens were used, with little or no documentation about the rationale for the choices of agents. We attempted to minimize selection bias by selecting cases and controls from the same population in a systematic manner. Recall bias may be present due to cases being more thoroughly questioned about risk factors than controls. This was true for travel history, with recorded responses for 51 % of cases and 16 % of controls. Conversely patients were more thoroughly questioned about recent antimicrobial use, with recorded responses for 73 % of cases and 92 % of controls. It is possible that patients who underwent TRUS biopsy at participating hospitals presented with infections elsewhere, although routine advice was given to patients to represent to Cabrini Hospital if complications developed as it is the only private hospital in this region with an emergency department. In addition the use of coding to identify cases is subject to inherent error in data capture and relevant patients may have consequently been missed. The absence of positive clinical isolates for all septic patients reflects the sensitivity of cultures as well as the use of prior antibiotics. The association between a higher number of cores and reduced sepsis is difficult to explain and is likely to reflect confounding by unknown factors.

## Conclusion

In this study TRUS biopsy sepsis was found to inflict a significant impact on patients and the healthcare system. A wide variety of different prophylaxis regimens and bowel preparation routines were recorded. Procedural variables such as extended prophylaxis regimens and the use of an enema did not show added benefit and may therefore be superfluous. This study also identified recent international travel and recent antimicrobial use as risk factors that were associated with sepsis. Patients with these factors may represent a better target population for intervention with alternative preventative strategies. These options include augmented prophylaxis, tailored prophylaxis or the transperineal biopy approach.
